# Do-it-yourself networks: a novel method of generating weighted networks

**DOI:** 10.1098/rsos.171227

**Published:** 2017-11-22

**Authors:** D. W. Shanafelt, K. R. Salau, J. A. Baggio

**Affiliations:** 1 Centre for Biodiversity Theory and Modelling, Theoretical and Experimental Ecology Station, CNRS and Paul Sabatier University, 09200 Moulis, France; 2 Department of Mathematics, The University of Arizona, 617 North Santa Rita Avenue, Tucson, AZ 85721, USA; 3 Department of Environment and Society, Utah State University, 5215 Old Main Hill, Logan, UT 84322, USA

**Keywords:** adjacency matrix, network and graph theory, optimization, weighted network

## Abstract

Network theory is finding applications in the life and social sciences for ecology, epidemiology, finance and social–ecological systems. While there are methods to generate specific types of networks, the broad literature is focused on generating *unweighted* networks. In this paper, we present a framework for generating *weighted* networks that satisfy user-defined criteria. Each criterion hierarchically defines a feature of the network and, in doing so, complements existing algorithms in the literature. We use a general example of ecological species dispersal to illustrate the method and provide open-source code for academic purposes.

## 1. Introduction

Network theory is gaining traction in the life and social sciences, finding applications in the fields of ecology, economics, epidemiology, finance and more recently, in analysing social–ecological systems [1–4]. In ecology, there is growing interest in the role of connectivity between spatial locations on the stability and resilience of those systems [5,6]. Numerous other applications exist for the study of infectious disease spread [7,8], optimal management of spatially distributed species [9], neural communication [10], bank failure [11] and the diffusion of ideas [12]. While there are methods to generate specific types of networks (table 1), these are primarily limited to *unweighted* networks [1]. In this paper, we present a general framework for generating suites of *weighted* networks that meet user-defined criteria, e.g. symmetry, clustering or other metrics measuring network connectivity [3,23]. The framework makes use of a hierarchy of metrics, where each metric specifies a distinct feature that describes the structural properties of the network; the hierarchy gives a natural order in which to consider the addition of new metrics. Our method uses optimization theory to create adjacency matrices that define the relationship between different entities such as spatial locations or the interactions between species, people and institutions. Our work is most akin to the network generation algorithms of Carayol *et al*. [15] and Small *et al*. [16], who use genetic algorithms and pay-off functions to optimize the generation process. However, we specifically generate *weighted* networks and optimize a suite of network metrics rather than node degree. We demonstrate our method using a general example of ecological species dispersal and provide open-source code of our algorithm for academic purposes.^1^

**Table 1.** Common network algorithms in the literature.

algorithm	brief summary	reference
Asano	Given a *k*-connected network of *N* nodes and a particular degree distribution. Order the nodes of the graph (*n_i_*) by decreasing degree such that *n*_1_ > *n*_2_ > … > *n_N_*. Recursively connect the nodes of the lowest degree (*h*) to those of the highest degree (from *n*_1_ to the first node of degree *h*, *n_x_*) and to those of the lowest degree (from *n_N_* to $n_{N-(h-x)}$).	Asano [13]
Bansal	Given a random network of *N* nodes. Iteratively select a random edge in the network and rewire it to a new node chosen in proportion to its degree. Continues rewiring until the coefficient of variation of the degree distribution is equal to one (characterizes an exponential degree distribution).	Bansal *et al*. [14]
Markov chain (maximum likelihood)	Given a network of *N* nodes. Iteratively add a new node or add, keep, or remove connections between nodes to maximize a pay-off function, such as the likelihood of reaching a particular node degree distribution or a net benefit/cost function of the connections between nodes.	Carayol *et al*. [15], Small *et al*. [16]
random	For each node *i* in a network of size *N*, there is a probability *p* that node *i* is connected to node *j*. Poisson degree distribution.	Erdős & Rényi [17], Gilbert [18], Newman *et al*. [19]
scale-free	Given an initial number of nodes in a network, *N*(0). For a set of *t* = 1,2, … ,*T* iterations, add a new node *n*(*t*) that is connected to no more than *N*(0) nodes in the system. The probability that the new node is connected to node *i* is given by $k_{i}/\Sigma_{j}k_{j}$, where *k_i_* is the number of connections of node *i*. Exhibits preferential connections between nodes. Degree distribution follows a power law.	Barabasi & Albert [20], Barabasi *et al*. [21]
small world (clustered)	Given a ring lattice network of *N* nodes with *k* connections per node. Rewire the network by reconnecting each edge of node *i* to a randomly chosen node *j* with probability *p*. Degree distribution follows a power law.	Watts & Strogatz [22]

## 2. Terminology

For the purposes of illustrating the algorithm, we consider a ‘habitat graph’ network composed of spatially distinct habitats or patches that are connected by species dispersing among them. However, our method is not limited to spatial networks and can be used to generate *weighted* networks in other applied fields such as those of species interaction or social networks. See [1,3] for reviews of different types of networks across disciplines.

In our study, each patch is referred to as a node; each connection is referred to as an edge, which allows for the flow of species between patches. An adjacency matrix describes the connections between patches.

Let us construct an adjacency matrix that represents the connectivity between *n* spatial locations or patches. Patches are connected by the movement of species, people or any flow between spatial locations. For illustrative purposes, we consider the case of a single species dispersing between patches.

Let the entries of the adjacency matrix correspond to the cost of movement between patches where the dispersal of species is symmetric, i.e. species move bidirectionally at the same rate. We assume a *weighted* network, where the off-diagonal entries of the adjacency matrix are constrained to be real numbers greater than or equal to zero. Increasing the cost of movement between patches to infinity is equivalent to assuming that two patches are not connected.^2^

Mathematically we define a randomly generated adjacency matrix describing the cost of species movement between patches as:

2.1 $$A=\left[\begin{array}{ccc} a_{11} & \cdots & a_{1n}\\ \vdots & \ddots & \vdots \\ a_{n1} & \cdots & a_{nn} \end{array}\right],$$

where *A* is a square *n* × *n* matrix and *a_ij_* is the degree of connectivity between patches *i* and *j*. Restrict *a_ij_* > 0 for all *i *≠ *j*. We assume that a patch cannot be connected to itself, i.e. *A* is a zero-diagonal matrix.^3^

We constrain the adjacency matrix to possess a spectral radius of *r*, along with a specific variance (*v*) and skewness (*s*) of the associated dominant eigenvector $\vec{x_{r}}$. We focus on these three for the following reasons. First, because we are primarily interested in *weighted* networks, metrics concerning node degree distributions (e.g. node or link density) are less appropriate. With *weighted* networks, our focus is the node connectivity distribution. Second, our metrics are particularly useful for illustrating a hierarchical approach to network construction and identification. Combining multiple network metrics—starting with general summary measures and then moving to more specific metrics (the hierarchy of ordering)—gives a more holistic understanding of network structure. For instance, spectral radius is a good proxy of connectivity [26] but there exist an infinite number of potential networks for a given spectral radius. Combining spectral radius with the dominant eigenvector provides a more complete picture of network structure. For any given spectral radius there exists a dominant eigenvector that can be described as a distribution of node connectivity scores. The statistical moments of such a distribution are a natural (hierarchical) method of discerning differences between networks. In other words, any two networks can be differentiated as higher statistical moments are calculated.

The spectral radius is the largest (dominant) eigenvalue of the adjacency matrix.^4^ In network terms, it represents the average distance it takes to traverse across the entire landscape and is therefore a proxy of network connectivity [26]. A low (high) spectral radius indicates a network that is highly (poorly) connected. We formally define the spectral radius as:

2.2 $$r=\text{max}\{\lambda_{1},\lambda_{2},\lambda_{3},\ldots ,\lambda_{n}\},$$

where ${\left\{\lambda_{k}\right\}}_{k=1\ldots n}$ are the eigenvalues associated with *A*. Let $\vec{x_{r}}$ represent the eigenvector associated with the dominant eigenvalue.

**Figure 1.**
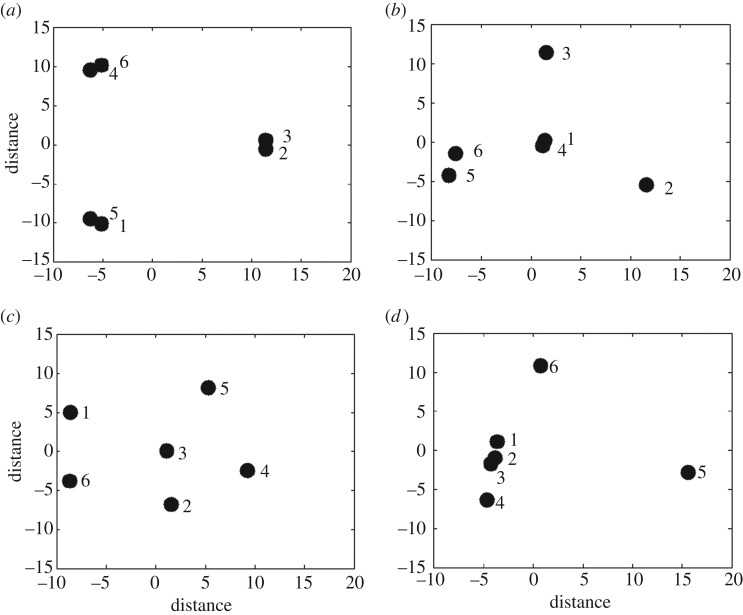
Comparison of networks with different properties. Note that each network has six nodes. (*a,b*) Networks have the same spectral radius (*r* = 80 km) but different variances (0 and 0.026). In *a*, all nodes contribute evenly to the overall connectivity of the network; in *b*, node contribution is not homogeneous. Nodes 1 and 4 are more connected than the others. (*c,d*) Networks have the same spectral radius and variance (*r* = 65 km, *v* = 0.0086) but different skewness (−1.79 and 1.086). In *c*, only node 3 is a strong contributor to connectivity; in *d*, nodes 1–4 contribute strongly. Adapted from Salau *et al*. [28].

Spectral radius alone is insufficient to guarantee a particular network structure. For a specific spectral radius there exists an infinite number of potential network configurations (figure 1). This makes it difficult to confidently generalize conclusions drawn from network process outcomes. Therefore, we further constrain the adjacency matrix (of spectral radius, *r*) to possess a defined variance and skewness in the associated dominant eigenvector, $\vec{x_{r}}$.

In a network context, the dominant eigenvector represents a collection of node connectivity scores or ‘eigenvector centralities’ of the network [29]. Each component in the dominant eigenvector measures the relative contribution of each node to the overall connectivity of the network. Each network consists of nodes with varying degrees of contribution. Some nodes may be high contributors to connectivity while other nodes may be isolated and contribute less. We define highly (weakly) connected nodes within a given network as nodes with a contribution score greater (less) than the mean contribution; where, the mean contribution score is the average of the dominant eigenvector components.

Variance of the dominant eigenvector measures the spread in node contribution across the network and thus quantifies node heterogeneity for a given network. In statistical terms, variance is the mean squared deviation of node contribution to connectivity. A low variance indicates that each node contributes similarly to network connectivity; a high variance is indicative of a wide distribution in node contribution.

Skewness of the dominant eigenvector measures the net proportion of highly to weakly connected nodes in a network. A skewness of zero implies an even proportion of highly and weakly connected nodes. A positive (negative) skewness indicates a greater (lower) number of highly connected to weakly connected nodes. Taken together, spectral radius, variance and skewness of a network highlight unique topological properties of the desired network (figure 1).

Variance (*v*) and skewness (*s*) of $\vec{x_{r}}$ take the forms:

2.3 $$v=\frac{1}{n}\overset{n}{\underset{k=1}{\sum }}{\left(x_{r,k}-\langle \vec{x_{r}}\rangle \right)}^{2}$$

and

2.4 $$s=\frac{(1/n)\sum_{k=1}^{n}{\left(x_{r,k}\right)}^{3}-3\langle \vec{x_{r}}\rangle v-{\left\langle \vec{x_{r}}\right\rangle }^{3}}{v^{3/2}},$$

where $x_{r,k}$ denotes the *k*th element of $\vec{x_{r}}$ and $\left\langle \vec{x_{r}}\right\rangle$ represents the mean of the elements of $\vec{x_{r}}$.

Alternatively, one may use other sets of network metrics to constrain the desired properties of the network. Many established network metrics are highly correlated but a clear description of how they hierarchically build upon each other is lacking in the literature [3,30]. By using the moments approach to summarize a network's node connectivity scores, each of our metrics highlights a distinct feature of networks and make hierarchy a non-issue [28].

## 3. The algorithm

In this section, we outline an algorithm to generate networks that conform to specific values for all three metrics [28]. Let r*, v* and s* serve as user-defined criteria that indicate the desired spectral radius and variance and skewness of the associated dominant eigenvector.^5^

We generate a random adjacency matrix, *A*_0_, to serve as initial conditions. Denote the spectral radius and variance/skewness of the associated eigenvector of *A*_0_ as *r*_0_, *v*_0_ and *s*_0_, respectively. In our algorithm, *A*_0_ and its associated network metrics are updated according to a numerical hill-climb method.

We choose the entries of *A*_0_ such that they minimize the weighted sum of squared differences between the properties of our ‘new’ adjacency matrix and our user-defined criteria.^6^ This results in the following minimization problem:

3.1 $$\text{mi}{\text{n}}_{a_{ij}}[\omega_{1}{\left(r^{*}-r_{0}\right)}^{2}+\omega_{2}{\left(v^{*}-v_{0}\right)}^{2}+\omega_{3}{\left(s^{*}-s_{0}\right)}^{2}].$$

The values of *ω_k_* are the weights associated with the preferred degree of accuracy of the estimated entry to the predefined user criteria. The larger the weight, the greater the variable contributes to the sum of squares and the closer the estimated value must be to the desired theoretical value. The values of *r*_0_, *v*_0_ and *s*_0_ are determined directly from the entries of *A*_0_ and are, therefore, functions of *a_ij_*.

The minimization problem in (3.1) is constrained by (subject to) a set of equations defining the structural properties of the desired adjacency matrix. The constraints form an *n*^2^ × *n*^2^ system of equations such that:

3.2 $$[B]\;\left[\begin{array}{c} a_{11}\\ \vdots \\ a_{nn} \end{array}\right]=[E].$$

Collectively, the above equation is referred to as the equality constraints. On the left-hand side, the matrix *B* refers to the boundary equality conditions matrix. The matrix *B* is an *n*^2^ × *n*^2^ matrix that captures the characteristics of the matrix *A*_0_. Each row corresponds to an equation describing the connectivity between patches. Each column corresponds to a variable representing an entry in *A*_0_. The matrix *B* is multiplied by an *n*^2^ × 1 vector of variables, one for each entry of *A*_0_. The solutions are given by an *n*^2^ × 1 vector *E*, often referred to as the equality conditions. Together (3.2) forms a system of at most *n*^2^ equations and *n*^2^ unknowns that capture the desired structural properties of the adjacency matrix. An illustration of the construction of (3.2) for a three-patch system is found in the electronic supplementary material, A. We present the general method below.

Initially define *B* and *E* to be a zero matrix and vector, respectively. For the entries of *B* corresponding to the *diagonal* and *zero off-diagonals* of *A*_0_, the following must hold:

— A patch cannot be connected to itself. For each diagonal entry of *A*_0_ there exists a row in *B* such that the corresponding entry of *a_ii_* in *B* is equal to one with all other entries in the row equal to zero. (After matrix multiplication in (3.2) this results in *a_ii_* = 0).
— To specify that two patches are not connected in the adjacency matrix, set the entries of *B* corresponding to the respective off-diagonal entries of *A*_0_ equal to zero. In other words, if *a_ij_* = *a_ji_* = 0 in *A*_0_, then there exist two rows in *B* such that the corresponding entries for *a_ij_* and *a_ji_* in *B* should be one while all other entries in both rows are equal to zero.

For the entries of *B* corresponding to the *non-zero off-diagonals* of *A*_0_, the following must hold:

— For each pair of connected patches in *A*_0_, there exists a row in *B* such that the corresponding entries for *a_ij_* and *a_ji_* are equal to 1 and −1, respectively. All other entries in the row are 0. This implies symmetry. (After matrix multiplication we obtain *a_ij_* = *a_ji_*.)

The minimized values of *A*_0_ are fed into a generic hill-climbing numerical algorithm to solve for the optimal solution. The pseudo-code for the method is outlined below:

— Define a new adjacency matrix consisting of the minimized values of *A*_0_ as $\tilde{A_{0}}$. Let the values of the network metrics associated with $\tilde{A_{0}}$ be given by $\tilde{r_{0}}$, $\tilde{v_{0}}$ and $\tilde{s_{0}}$.
— Compare the values of each network metric derived from $\tilde{A_{0}}$ to r*, v* and s*. One method to do so is to calculate the absolute value of the difference between the desired and derived metrics (e.g. $\left|r^{*}-\tilde{r_{0}}\right|$, $\left|v^{*}-\tilde{v_{0}}\right|$, $\left|s^{*}-\tilde{s_{0}}\right|$). Another is to calculate the per cent difference between the desired and derived metrics.
— If all three metric differences fall below a pre-determined tolerance level, the algorithm concludes.
— However, if the difference between any of the metrics exceeds a pre-determined tolerance level:
  (i) Randomize one off-diagonal element of $\tilde{A_{0}}$ subject to the assumptions discussed above.
  (ii) Define the perturbed $\tilde{A_{0}}$ as new initial conditions for the minimization problem in (3.2). In other words, update *A*_0_, *r*_0_, *v*_0_ and *s*_0_ to be the perturbed adjacency matrix $\tilde{A_{0}}$. Minimize the sum of squared differences.
  (iii) Update $\tilde{A_{0}}$.

**Figure 2.**
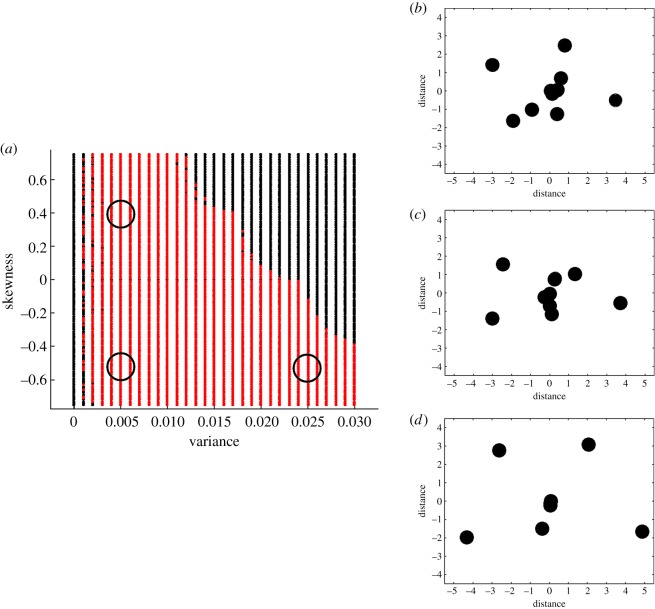
Available network configurations that can be generated using the algorithm. Each network consists of 10 patches. (*a*) Each combination of variance (v*) and skewness (s*) of the dominant eigenvector assumes a desired spectral radius (λ*) of 20. The minimum (*w*_min_) and maximum (*w*_max_) distance between nodes are set to 1 and 50, respectively. A red dot indicates convergence; a black dot indicates that the algorithm did not converge to a solution. Other potential configurations can be found in electronic supplementary material, A. (*b–d*) Visual representation of the networks indicated by circles in *a*, where: v* = 0.005, s* = 0.4 (*b*); v* = 0.005, s* = −0.5 (*c*); and v* = 0.025, s* = −0.5 (*d*).

The resulting output is an *n* × *n* adjacency matrix that is symmetric and possesses a spectral radius, variance and skewness of its associated eigenvector of r*, v* and s*. Figure 2 illustrates available network configurations generated using our method. Other configurations are found in electronic supplementary material, A, including network configurations of 50, 100 and 250 patches. While small compared with neural networks or the World Wide Web, these network sizes are suitable for many empirical social and ecological networks [4,31–33].

Convergence of the algorithm is dependent on several factors. First, for a given spectral radius and number of patches, there exists a lower and upper bound on the feasible values of the variance and skewness of the dominant eigenvector. These are derived in electronic supplementary material, A. Second, while minimizing the weighted sum of squared differences guarantees a concave function, the value of the desired properties of the adjacency matrix (and their associated weights) in (3.1) determine the specific shape of the function. The flatter the function to be minimized, the more difficult it is for the algorithm to converge to a solution. Finally, we cannot rule out the possibility of convergence to a local minima, in contrast with a global minima. However, this is a general problem characteristic of optimization theory [34,35], and formal analysis is left for future work.

Our algorithm was coded in Matlab 2016a. Source code is available in the electronic supplemental material and is free for academic use.^7^

## 4. Discussion

By varying initial conditions one may generate an infinite number of *weighted* networks with specific structural properties. Defining multiple criteria in the generation process allows one to fine-tune the structure of the resulting network, and explicitly test the effect of network structure on system properties such as stability or resilience.

Further, our method is not limited to spectral radius and eigenvector centrality, but may be applied using any network metric deemed appropriate to describe the connectivity of the system. For example, it is possible to use clustering and other metrics that are employed to analyse social and biological networks (i.e. clustering, degree centrality and betweenness) [1,2,22]. It is feasible to use our algorithm to generate networks with combinations of distances between nodes, node contributions to connectivity and/or clustering.

Nor is our method limited to symmetric, bidirectional dispersal. By changing the system of equations in (3.2), one may allow for unidirectional or asymmetric dispersal between patches. The method can also be extended to multiple species and *unweighted* networks. For the former, each species has its own adjacency matrix that describes the flow of that species within the network. The latter is intrinsically more difficult. Intuitively one would constrain the entries of the adjacency matrix to zero or one, and (if desired) solve for an adjacency matrix with a particular average, variance and skewness in the number of connections per node. In this case, the minimization problem in (3.1) becomes a nonlinear binary integer problem, which is difficult to solve and is left for future work.^8^

Like other network generation methods (table 1), there is the possibility that the networks we generate reflect only a small subset of the entire suite of networks that meet our specified criteria. While we know of no way to explicitly test this, we can combine our method for generating *weighted* networks with those of *unweighted* networks to more fully ‘paint the picture’ of a network's structure. We may, for example, create a *weighted* network that has a particular node degree distribution. To do so, one would first generate an *unweighted* network describing the structural connections between nodes (e.g Erdõs-Réyni [17], scale-free [20], small world [22]). This would have a particular node degree distribution. The binary connected/not connected coefficients could then be fed into the equality constraints of (3.2) to create a weighted network with a specific degree distribution that also meets the desired properties of the weights.

Our method has applications across disciplines. We briefly discuss several examples. In epidemiology, disease propagation is often faster in structured population networks than random ones [20]. More specifically, the structure of the network of contacts has implications for the management of human and animal disease spread [8,36]. In ecology, the dispersal of species plays a distinct role in the persistence and survival of species. Examples include mass and rescue effects [37,38], source–sink dynamics [39,40] and spatial insurance [41,42]. At the core of each of these principles is the underlying structure describing the movement of species between spatial locations. More recent work has identified ‘keystone patches’—patches that contribute strongly to diversity or stability of the entire system [43]. Finally, the management of spatially distributed resources has been a topic in the field of economics [44]. Though much of the literature focuses on fisheries management [9,44,45], applications exist for the spatial management of other renewable resources [46] and invasive species [47].

While we focus the application of our method on spatial networks, it is by no means limited to them. Our method is suitable for generating matrices of species interactions such as competition, parasitism or mutualism. In ecology, one common measure of ecosystem stability is the dominant eigenvalue of the interaction matrix or ‘asymptotic resilience’ [48]. A negative (positive) value indicates a stable (unstable) system, with the magnitude being a loose indicator of how stable the system is. Recent advances have demonstrated that for random interaction matrices the dominant eigenvalue can be a poor indicator of stability compared to other metrics [49]. It would be useful to explicitly test under which species interaction structures the dominant eigenvalue is a good or poor indicator of stability.

Our method complements existing algorithms for generating networks (table 1) while giving researchers precision in the structure of networks they wish to construct. We hope that our algorithm will aid researchers in testing the effects of explicit network structure on model outcomes.

## Supplementary Material

Supplemental Material A. Bounding the variance and skewness of the dominant eigenvalue, illustration of the equality constraints, and additional network configurations

## Supplementary Material

Supplemental Material B. Algorithm source code

## Footnotes

1 Source code and convergence data can be retrieved from the Open Science Framework (https://osf.io/4sd65).

2 This is in contrast with an *unweighted* network where the off-diagonal entries of the adjacency matrix are constrained to be one or zero—i.e. patches either are or are not connected. Unweighted networks often reflect the structural connections between patches. Weighted networks contain additional information such as distance between patches, patch size and quality, or the probability of dispersal between patches [3,24]. In our case, the weights correspond to the cost of movement between patches. By setting a threshold to determine if a pair of nodes are connected or not, one can convert weighted networks into unweighted ones [3,25].

3 Conditional on the model framework, the diagonals of the adjacency matrix do not necessarily need to equal zero. For example, if one was concerned with species interactions or *unweighted* spatial networks, then the diagonals of the adjacency matrix could correspond to the growth rate(s) of species.

4 Because we assume a *weighted* network, the off-diagonal of the adjacency matrix are composed of positive real numbers. Therefore, we are guaranteed at least one real, positive eigenvalue where the elements in the associated eigenvector are strictly positive [27]. Alternatively, one may use other derivations of spectral radius, which are equivalent for *weighted* and *unweighted* networks [28].

5 For some network with a given number of patches (*n*) and spectral radius (*r**), it follows that bounds for the variance and skewness of the associated eigenvector are given by:

$$v^{*}\leq \frac{1}{n}-{\left[\left(\frac{n-1}{n}\right)\left(\frac{w_{\min}}{r^{*}}\right)\right]}^{2}$$

$$\frac{-3g(v)v^{*}-{\left[g(v)\right]}^{3}}{{\left(v^{*}\right)}^{3/2}}<s^{*}<\frac{(1/n)-3g(v)v^{*}-{\left[g(v)\right]}^{3}}{{\left(v^{*}\right)}^{3/2}}$$

where *w*_min_ is the user-defined minimum value of an entry of *A*_0_. The function *g*(*v*) represents the mean of the dominant eigenvector, which exhibits a one-to-one relationship with the variance where $g(v)={\left((1/n)-v^{*}\right)}^{1/2}$ [28]. These inequalities represent ‘weak’ bounds for *v** and *s**. Proofs for these inequalities are found in electronic supplementary material, A. Examples of available network configurations generated using our method can be found in figure 2; electronic supplementary material, A.

6 The sum of squared differences is a well-known method to compare theoretical and predicted outcomes in ecology and statistics. Further, because it is squared, it guarantees concavity.

7 The algorithm was coded in Matlab for applicability and readability to a broad audience. Computational efficiency could be increased by coding the algorithm in another platform such as C++ or Python, allowing for the generation of larger networks. Explicitly accounting for the assumptions of the desired adjacency matrix will also improve computation efficiency. For example, by imposing symmetry in (3.2) one reduces the number of unknown variables by half.

8 Alternatively, one could generate a weighted network with specific properties and convert it to an unweighted network by assigning a weight threshold that defines the presence or absence of a connection [3,25].

## Data accessibility

We include source code for the algorithm in electronic supplementary material, B. Alternatively, source code and convergence data can be retrieved from the Open Science Framework (https://osf.io/4sd65).

## References

[1] Albert R, Barabasi A 2002 Statistical mechanics of complex networks. Rev. Modern Phys. 74, 47–97. (doi:10.1103/RevModPhys.74.47)

[2] Newman MEJ 2003 The structure and function of complex networks. SIAM Rev. 45, 167–256. (doi:10.1137/S003614450342480)

[3] Rayfield B, Fortin M, Fall A 2011 Connectivity for conservation: a framework to classify network measures. Ecology 92, 847–858. (doi:10.1890/09-2190.1) 21661548 10.1890/09-2190.1

[4] Baggio JA, Burnsilver S, Arenas A, Magdanz J, Kofinas G, DeDomenico M 2016 Multiplex social ecological network analysis reveals how social changes affect community robustness more than resource depletion. Proc. Natl Acad. Sci. USA 113, 13 708–13 713. (doi:10.1073/pnas.1604401113) 10.1073/pnas.1604401113 PMC5137762 27856752

[5] Urban DL, Minor ES, Tremi E, Schick RS 2009 Graph models of habitat mosaics. Ecol. Lett. 12, 260–273. (doi:10.1111/j.1461-0248.2008.01271.x) 19161432 10.1111/j.1461-0248.2008.01271.x

[6] Loreau M 2010 From populations to ecosystems: theoretical foundations for a new ecological synthesis. Princeton, NJ: Princeton University Press.

[7] May RM 2006 Network structure and the biology of populations. Trends Ecol. Evol. 21, 394–399. (doi:10.1016/j.tree.2006.03.013) 16815438 10.1016/j.tree.2006.03.013

[8] Ames GM, George DB, Hampson CP, Kanarek AR, McBee CD, Lockwood DR, Achter JD, Webb CT 2011 Using network properties to predict disease dynamics on human contact networks. Proc. R. Soc. B 278, 3544–3550. (doi:10.1098/rspb.2011.0290) 10.1098/rspb.2011.0290 PMC3189367 21525056

[9] Sanchirico JN, Wilen J 1999 Bioeconomics of spatial exploitation in a patchy environment. J. Environ. Econ. Manage. 37, 129–150. (doi:10.1006/jeem.1998.1060)

[10] Laughlin SB, Sejnowski TJ 2003 Communication in neuronal networks. Science 301, 1870–1874. (doi:10.1126/science.1089662) 14512617 10.1126/science.1089662 PMC2930149

[11] Allen F, Gale D 2000 Financial contagion. J. Polit. Economy 108, 1–33. (doi:10.1086/262109)

[12] Watts DJ 2002 A simple model of global cascades on random networks. Proc. Natl Acad. Sci. USA 99, 5766–5771. (doi:10.1073/pnas.082090499) 16578874 10.1073/pnas.082090499 PMC122850

[13] Asano T 1995 An *O*(*n*-log-log-*n*) time algorithm for constructing a graph of maximum connectivity with prescribed degrees. J. Comput. Syst. Sci. 51, 503–510. (doi:10.1006/jcss.1995.1086)

[14] Bansal S, Grenfell BT, Meyers LA 2007 When individual behaviour matters: homogeneous and network models in epidemiology. J. R. Soc. Interface 4, 879–891. (doi:10.1098/rsif.2007.1100) 17640863 10.1098/rsif.2007.1100 PMC2394553

[15] Carayol N, Roux P, Yildizoglu M 2008 Inefficiencies in a model of spatial networks formation with positive externalities. J. Econ. Behav. Organ. 67, 495–511. (doi:10.1016/j.jebo.2007.04.004)

[16] Small M, Li Y, Stemler T, Judd K 2015 Growing optimal scale-free networks via likelihood. Phys. Rev. E 91, 042801 (doi:10.1103/PhysRevE.91.042801) 10.1103/PhysRevE.91.042801 25974541

[17] Erdős P, Rényi A 1959 On random graphs I. Publ. Math. 6, 290–297.

[18] Gilbert EN 1959 Random graphs. Ann. Math. Stat. 30, 1141–1144. (doi:10.1214/aoms/1177706098)

[19] Newman MEJ, Strogatz SH, Watts DJ 2001 Random graphs with arbitrary degree distributions and their applications. Phys. Rev. E 64, 026118 (doi:10.1103/PhysRevE.64.026118) 10.1103/PhysRevE.64.026118 11497662

[20] Barabasi A, Albert R 1999 Emergence of scaling in random networks. Science 286, 509–512. (doi:10.1126/science.286.5439.509) 10521342 10.1126/science.286.5439.509

[21] Barabasi A, Albert R, Jeong H 1999 Mean-field theory for scale-free random networks. Physica A 272, 173–187. (doi:10.1016/S0378-4371(99)00291-5)

[22] Watts DJ, Strogatz SH 1998 Collective dynamics of 'small-world' networks. Nature 393, 440–442. (doi:10.1038/30918) 9623998 10.1038/30918

[23] Baggio JA, Salau K, Janssen MA, Schoon ML, Bodin O 2011 Landscape connectivity and predator-prey population dynamics. Landscape Ecol. 26, 33–45. (doi:10.1007/s10980-010-9493-y)

[24] Minor ES, Urban DL 2008 A graph-theory framework for evaluating landscape connectivity and conservation planning. Conserv. Biol. 22, 297–307. (doi:10.1111/j.1523-1739.2007.00871.x) 18241238 10.1111/j.1523-1739.2007.00871.x

[25] Thompson PL, Rayfield B, Gonzalez A 2017 Loss of habitat and connectivity erodes species diversity, ecosystem functioning, and stability in metacommunity networks. Ecography 40, 98–108. (doi:10.1111/ecog.02558)

[26] Jacobi MN, Jonsson PR 2011 Optimal networks of nature reserves can be found through eigenvalue perturbation theory of the connectivity matrix. Ecol. Appl. 21, 1861–1870. (doi:10.1890/10-0915.1) 21830724 10.1890/10-0915.1

[27] Caswell H 2001 Matrix population models: construction, analysis, and interpretation. Sunderland, MA: Sinauer Associates.

[28] Salau KR, Baggio JA, Janssen MA, Abbott JK, Fenichel EP 2015 Taking a moment to measure networks: a hierarchical approach. (http://arxiv.org/abs/1509.07813).

[29] Borgatti SP 2005 Centrality and network flow. Soc. Networks 27, 55–71. (doi:10.1016/j.socnet.2004.11.008)

[30] Estrada E, Bodin O 2008 Using network centrality measures to manage landscape connectivity. Ecol. Appl. 18, 1810–1825. (doi:10.1890/07-1419.1) 18839774 10.1890/07-1419.1

[31] Shigesada N, Kawasaki K 1997 Biological invasions: theory and practice. Oxford, UK: Oxford University Press.

[32] Hanski I 1999 Metapopulation ecology. Oxford, UK: Oxford University Press.

[33] Memmott J 1999 The structure of a plant-pollinator food web. Ecol. Lett. 2, 276–280. (doi:10.1046/j.1461-0248.1999.00087.x) 10.1046/j.1461-0248.1999.00087.x 33810635

[34] Kamien MI, Schwartz NL 1991 Dynamic optimization: the calculus of variations and optimal control in economics and management. New York, NY: Elsevier Science Publishing Co.

[35] Caputo MR 2005 Foundations of dynamic economic analysis: optimal control theory and applications. Cambridge, UK: Cambridge University Press.

[36] Shirley MDF, Rushton SP 2005 The impacts of network topology on disease spread. Ecol. Complex. 2, 287–299. (doi:10.1016/j.ecocom.2005.04.005)

[37] Brown JH, Kodric-Brown A 1977 Turnover rates in insular biogeography: effect of immigration on extinction. Ecology 58, 445–449. (doi:10.2307/1935620)

[38] Shmida A, Wilson MV 1985 Biological determinants of species diversity. J. Biogeogr. 12, 1–20. (doi:10.2307/2845026)

[39] Holt RD 1985 Population dynamics in two-patch environments: some anomalous consequences of an optimal habitat distribution. Theor. Popul. Biol. 28, 181–208. (doi:10.1016/0040-5809(85)90027-9)

[40] Pulliam HR 1988 Sources, sinks, and population regulation. Am. Nat. 132, 652–661. (doi:10.1086/284880)

[41] Yachi S, Loreau M 1999 Biodiversity and ecosystem productivity in a fluctuating environment: the insurance hypothesis. Proc. Natl Acad. Sci. USA 96, 1463–1468. (doi:10.1073/pnas.96.4.1463) 9990046 10.1073/pnas.96.4.1463 PMC15485

[42] Loreau M, Mouquet N, Gonzalez A 2003 Biodiversity as spatial insurance in heterogeneous landscapes. Proc. Natl Acad. Sci. USA 100, 12 765–12 770. (doi:10.1073/pnas.2235465100) 10.1073/pnas.2235465100 PMC240692 14569008

[43] Mouquet N, Gravel D, Massol F, Calcagno V 2013 Extending the concept of keystone species to communities and ecosystems. Ecol. Lett. 16, 1–8. (doi:10.1111/ele.12014) 10.1111/ele.12014 23062191

[44] Clark CW 2010 Mathematical bioeconomics: the mathematics of conservation, 3rd edn Hoboken, NJ: John Wiley & Sons, Inc.

[45] Brown G, Roughgarden J 1997 A metapopulation model with private property and a common pool. Ecol. Econ. 22, 65–71. (doi:10.1016/S0921-8009(97)00564-8)

[46] Smith MD, Sanchirico JN, Wilen JE 2009 The economics of spatial-dynamic processes: applications to renewable resources. J. Environ. Econ. Manage. 57, 104–121. (doi:10.1016/j.jeem.2008.08.001)

[47] Epanchin-Niell RS, Wilen JE 2012 Optimal spatial control of biological invasions. J. Environ. Econ. Manage. 63, 260–270. (doi:10.1016/j.jeem.2011.10.003)

[48] May RM 1973 Stability and complexity in model ecosystems. Princeton, NJ: Princeton University Press.

[49] Arnoldi J-F, Loreau M, Haegeman B 2016 Resilience, reactivity and variability: a mathematical comparison of ecological stability measures. J. Theor. Biol. 389, 47–59. (doi:10.1016/j.jtbi.2015.10.012) 26542944 10.1016/j.jtbi.2015.10.012

