# Supplementary material for: Do-it-yourself networks: a novel method of generating weighted networks
Source: R Soc Open Sci. 2017 Nov 22;4(11):171227. doi: 10.1098/rsos.171227 (PMC5717683; doi:10.1098/rsos.171227)
Supplement: Supplemental Material A. Bounding the variance and skewness of the dominant eigenvalue, illustration of the equality constraints, and additional network configurations [file rsos171227supp1.docx]

*Supplemental material A*

*Do-it-yourself (DIY) networks: A novel method of generating weighted networks*

David W. Shanafelt

Kehinde R. Salau

Jacopo A. Baggio

BOUNDING THE VARIANCE AND SKEWNESS OF A DOMINANT EIGENVECTOR

The following proofs detail how to bound the variance and skewness of an eigenvector centrality using the known quantities of the *DIY* network optimization problem. The known inputs include the number of nodes in the corresponding network (*n*), the spectral radius of the network ($r$), and the minimum value that edge weights may take in the system ($w_{min}$).

For our framework, we assume the existence of a positive spectral radius. In our example of a habitat graph, specifically we assume that the adjacency matrix is a square matrix with positive off-diagonals. See [Horn and Johnson (1990](#_ENREF_3)) and [Caswell (2001](#_ENREF_2)) for examples of other types of non-negative matrices that satisfy the structural requirements for a positive dominant eigenvalue.

In many networks of interest $w_{min}$ takes a value of zero, but in some systems a positive lower bound exists for differentiating between 2 nodes (see [Salau et al. (2015](#_ENREF_4)) for an ecological example). In the latter case, $w_{min}$ characterizes a stronger bounding for variance and, subsequently, skewness of an eigenvector centrality.

*Proposition 1: The network node number* (*n*), *spectral radius* ($r$), *and minimum node weight* ($w_{min}$) *characterize an upper bound for variance* ($v_{max}$) *of the dominant eigenvector* ($\boldsymbol{x}_{r}$).

*Proof*. Variance always has a lower bound at zero, so we focus on characterizing an upper bound. We calculate the strength of node $i$, written as $w_{i}$, as the sum of the weights on its connecting edges,

(S1) $w_{i}= \sum_{j} w_{i,j}$

where $w_{i,j}$ is the weight of the edge connecting nodes $i$ and $j$. One formula - provided by Frobenius ([Cao 1998](#_ENREF_1)) - for the spectral radius (*r*) of an adjacency matrix *A*, where $A=\left\{ w_{i,j} \right\}$, is,

(S2) $r= \frac{\sum_{i} \sum_{j} w_{i,j}x_{j}}{\sum_{k} x_{k}}=\frac{\sum_{i,j} w_{i,j}x_{j}}{\sum_{k} x_{k}}=\frac{\sum_{j} w_{j}x_{j}}{\sum_{k} x_{k}}$

where $x_{j}\in\boldsymbol{x}_{r}$ represents an element of the dominant eigenvector ($\boldsymbol{x}_{r}$) associated with the spectral radius. When a matrix possesses a positive dominant eigenvalue, the elements of the associated eigenvector are also positive ([Caswell 2001](#_ENREF_2)). Note that for weighted and unweighted networks with no isolated nodes, Equation S2 is equivalent to the value of the dominant eigenvalue ([Salau et al. 2015](#_ENREF_4)). We return to Equation S2 later in the proof.

[Salau et al. (2015](#_ENREF_4)) derive a one-to-one mapping between the mean ($\mu$) and variance ($v$) of the dominant eigenvector ($\boldsymbol{x}_{r}$). This relationship is based on the fact that the elements of $\boldsymbol{x}_{r}$ are always renormalized such that $\sum_{j=1}^{n} {x_{j}}^{2}=1$, which also implies that $x_{j}<1$ for all $j\in\left\{ 1,2,\ldots,n \right\}$. This one-to-one mapping forms the basis for the bounding relationship expressed below,

(S3) $v=\frac{1}{n}-\mu^{2}$ ([Salau et al. 2015](#_ENREF_4))

$=\frac{1}{n}-\left( \frac{\sum_{i} x_{i}}{n} \right)^{2}$

$=\frac{1}{n}-\left( \frac{\sum_{j} w_{j}x_{j}}{rN} \right)^{2}$ (Using Equation S2)

$\leq\frac{1}{n}-\left( \frac{\sum_{j} w_{j}{x_{j}}^{2}}{rn} \right)^{2}$

Since $\sum_{j=1}^{n} {x_{j}}^{2}=1$, $\sum_{j} w_{j}{x_{j}}^{2}$ is the rescaled mean of the set of $w_{j}$. Since the rescaled mean of a set of values will always be greater than or equal to the smallest value in that set, it follows that,

(S4) $\sum_{j} w_{j}{x_{j}}^{2}\geq\min_{j} w_{j}=\min_{j} \sum_{k} w_{j,k}\geq w_{min}\left( n-1 \right)$

The inequality in Equation S4 indicates that the rescaled mean weight of a network node is at most as small as a hypothetical node in the network that has only minimum weight connections with other nodes. Using Equation S4 we can simplify Equation S3 to,

(S5) $v_{max}\leq\frac{1}{n}-\left( \frac{w_{min}\left( n-1 \right)}{rn} \right)^{2}=\frac{1}{n}-\left[ \left( \frac{n-1}{n} \right)\left( \frac{w_{min}}{r} \right) \right]^{2}$

In sum, we now have an upper bound for the variance ($v_{max}$) of an eigenvector characterized entirely of the known inputs: number of network nodes (*n*), the desired spectral radius (*r*), and the minimum edge weight between any two nodes (*w_min_*).

*Proposition 2: The network node number* (*n*) and *variance* (*v*) *characterize upper and lower bounds for skewness* (*s*) *of the dominant eigenvector* ($\boldsymbol{x}_{r}$).

*Proof*. One representation for skewness of the dominant eigenvector is ([Salau et al. 2015](#_ENREF_4)),

(S6) $\mathbf{s=}\frac{\mathbf{E}\left( {\mathbf{x}_{\mathbf{r}}}^{\mathbf{3}} \right)\boldsymbol{-3\mu}\boldsymbol{\sigma}^{\mathbf{2}}\mathbf{-}\boldsymbol{\mu}^{\mathbf{3}}}{\boldsymbol{\sigma}^{\mathbf{3}}}\mathbf{=}\frac{\mathbf{E}\left( {\mathbf{x}_{\mathbf{r}}}^{\mathbf{3}} \right)\mathbf{-3g}\left( \mathbf{v} \right)\mathbf{v-}\left[ \mathbf{g}\left( \mathbf{v} \right) \right]^{\mathbf{3}}}{\mathbf{v}^{\mathbf{3}/\mathbf{2}}}$

where $\boldsymbol{v=\sigma}^{\boldsymbol{2}}$. We write $\boldsymbol{\mu=g}\left( \boldsymbol{v} \right)$ in Equation S6 to remind readers of the one-to-one relationship between the mean and variance of the dominant eigenvector due to renormalization of the elements of $\boldsymbol{x}_{\boldsymbol{r}}$ (Proposition 1). Skewness ($\boldsymbol{s}$) is almost entirely determined by variance ($\boldsymbol{v}$), which is a known input of the network optimization problem. We now use properties of the dominant eigenvector to bound the expected value term in Equation S6.

In Proposition 1, we wrote that the elements of $\boldsymbol{x}_{r}$ are always renormalized such that $\sum_{j=1}^{N} {x_{j}}^{2}=1$, which implied that $x_{j}<1$ for all $j\in\left\{ 1,2,\ldots N \right\}$. It follows that $\sum_{j=1}^{N} {x_{j}}^{3}<1$ must also hold. Thus, a lower and upper bound for the expected value term in Equation S6 is,

(S7) $0<\mathbf{E}\left( {\mathbf{x}_{\mathbf{r}}}^{\mathbf{3}} \right)=\frac{\sum_{j} {x_{j}}^{3}}{n}<\frac{1}{n}$

Recall that we assume the existence of a positive dominant eigenvalue. Thus, the elements of the associated eigenvector are strictly positive and the cubed expected value in S7 must be greater than zero.

Using Equation S7, a lower bound and upper bound for skewness is,

(S8) $\frac{\boldsymbol{-3}\boldsymbol{g}\left( \boldsymbol{v} \right)\boldsymbol{v-}\left[ \boldsymbol{g}\left( \boldsymbol{v} \right) \right]^{\boldsymbol{3}}}{\boldsymbol{v}^{\boldsymbol{3}/\boldsymbol{2}}}\boldsymbol{<}s\boldsymbol{<}\frac{\frac{\boldsymbol{1}}{\boldsymbol{n}}\boldsymbol{- 3}\boldsymbol{g}\left( \boldsymbol{v} \right)\boldsymbol{v -}\left[ \boldsymbol{g}\left( \boldsymbol{v} \right) \right]^{\boldsymbol{3}}}{\boldsymbol{v}^{\boldsymbol{3}/\boldsymbol{2}}}$

where $g\left( v \right)=\left( \left( 1/n \right)-v \right)^{1/2}$. In sum, Equation S8 represents a lower and upper bound for the skewness (*s*) of an eigenvector characterized entirely of the known inputs: number of network nodes (*n*) and variance (*v*).

ILLUSTRATION FOR THE CONSTRUCTION OF THE EQUALITY CONSTRAINTS

Define an adjacency matrix, $A_{0}$, to serve as initial conditions such that,

(S9) $A_{0}=\left[ \begin{matrix} a_{11} & a_{12} & a_{13} \\ a_{21} & a_{22} & a_{23} \\ a_{31} & a_{32} & a_{33} \end{matrix} \right]$

where $a_{ij}$ represents the cost of movement between patches $i$ and $j$. Note that $A_{0}$ represents a three patch system ($n=3$). The values of spectral radius ($r_{0}$), variance ($v_{0}$) and skewness ($s_{0}$) of the associated eigenvector are determined directly from $A_{0}$ and are functions of $a_{ij}$.

Assuming *symmetric*, *bidirectional* dispersal, the equality constraints are given by,

(S10) $\left[ B \right]\left[ \begin{matrix} a_{11} \\ \vdots\\ a_{nn} \end{matrix} \right]=\left[ E \right]$

(S11) $\left[ \begin{matrix} 1 & 0 & 0 & 0 & 0 & 0 & 0 & 0 & 0 \\ 0 & 0 & 0 & 0 & 1 & 0 & 0 & 0 & 0 \\ 0 & 0 & 0 & 0 & 0 & 0 & 0 & 0 & 1 \\ 0 & 1 & 0 & -1 & 0 & 0 & 0 & 0 & 0 \\ 0 & 0 & 1 & 0 & 0 & 0 & -1 & 0 & 0 \\ 0 & 0 & 0 & 0 & 0 & 1 & 0 & -1 & 0 \end{matrix} \right]\left[ \begin{matrix} a_{11} \\ \begin{matrix} a_{12} \\ \begin{matrix} a_{13} \\ \begin{matrix} a_{21} \\ \begin{matrix} a_{22} \\ a_{23} \\ a_{31} \\ a_{32} \\ a_{33} \end{matrix} \end{matrix} \end{matrix} \end{matrix} \end{matrix} \right]=\left[ \begin{matrix} 0 \\ 0 \\ \begin{matrix} 0 \\ 0 \\ 0 \\ 0 \end{matrix} \end{matrix} \right]$

where the bracketed components of equation S11 correspond to those in equation S10. The system of equations in (S10) and (S11) are equivalent to,

(S12) $a_{11}=a_{22}=a_{33}=0$

(S13) $a_{12}=a_{21}$

(S14) $a_{13}=a_{31}$

(S15) $a_{23}=a_{32}$

where (S12) indicates zero diagonals and (S13)-(S15) imply symmetry.

ADDITIONAL NETWORK CONFIGURATIONS


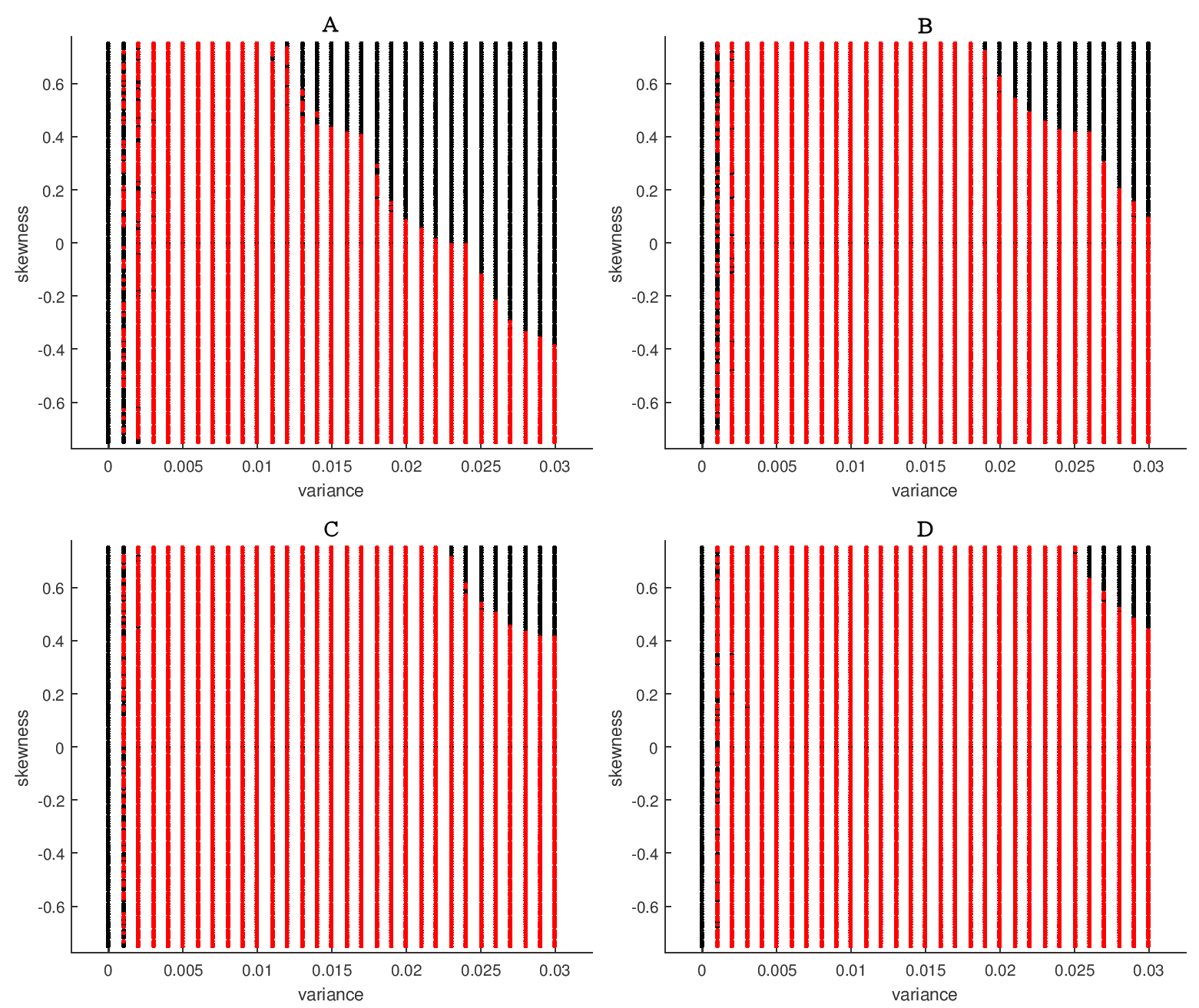


Figure S1. Additional available network configurations that can be generated using the algorithm. Each network consists of ten patches. Each combination of variance ($v^{*}$) and skewness ($s^{*}$) of the dominant eigenvector assumes a desired spectral radius ($\lambda^{*}$) of 20 (A), 30 (B), 40 (C), and 50 (D). The minimum ($w_{min}$) and maximum ($w_{max}$) distance between nodes are set to 1 and 50 respectively. A red dot indicates convergence; a black dot indicates that the algorithm did not converge to a solution.


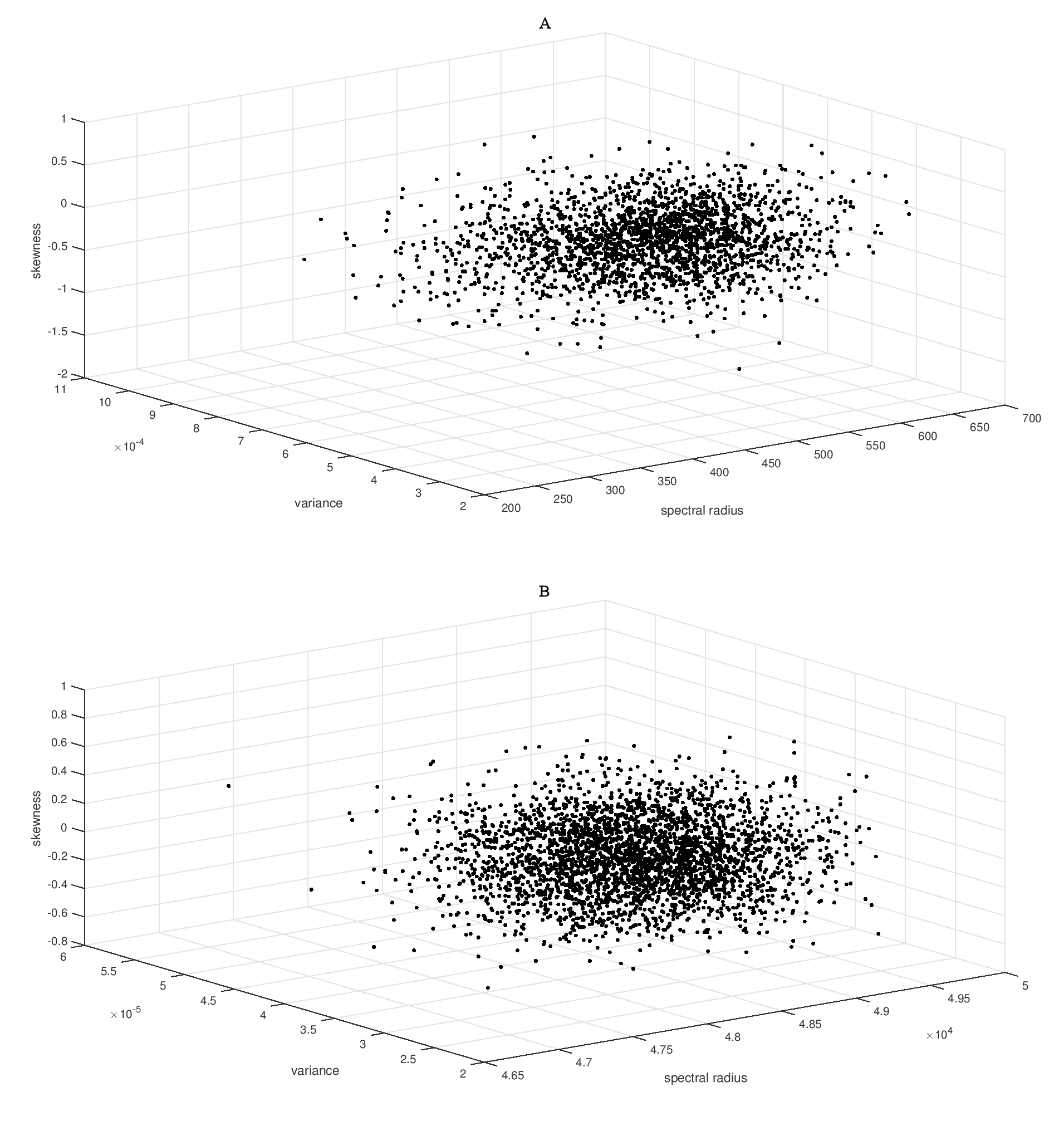


Figure S2. Available network configurations for larger networks of 50 (A) and 100 (B) patches. Each dot represents a plausible combination of spectral radius, and variance and skewness of the dominant eigenvector. Data were generated to demonstrate the applicability of the method to larger networks, not to create specific configurations. Altering the desired spectral radius and variance and skewness of the associated eigenvector will change the distribution of the network properties.


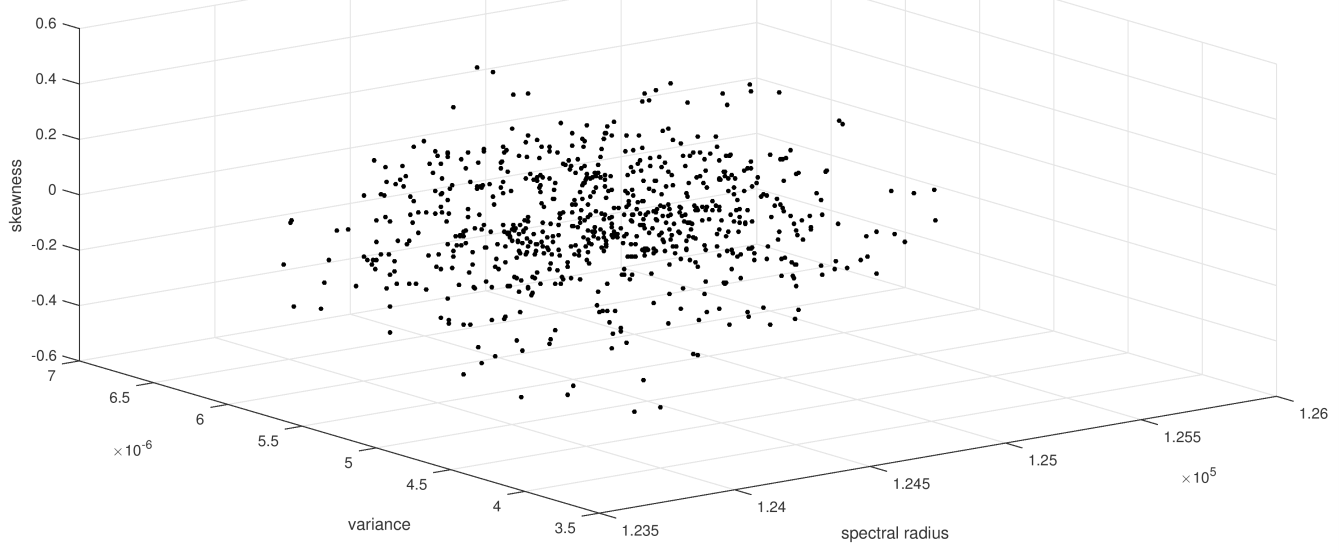


Figure S3. Available network configurations for networks of 250 patches. Each dot represents a plausible combination of spectral radius, and variance and skewness of the dominant eigenvector. Data were generated to demonstrate the applicability of the method to larger networks, not to create specific configurations. Altering the desired spectral radius and variance and skewness of the associated eigenvector will change the distribution of the network properties.

REFERENCES

Cao, D. 1998. Bounds on eigenvalues and chromatic numbers. Linear Algebra and Its Applications **270**:1-13.

Caswell, H. 2001. Matrix population models: Construction, analysis, and interpretation. Sinauer Associates, Sunderland, Massachusetts.

Horn, R. A. and C. R. Johnson. 1990. Matrix analysis. Cambridge University Press, Cambridge.

Salau, K. R., J. A. Baggio, M. A. Janssen, J. K. Abbott, and E. P. Fenichel. 2015. Taking a moment to measure networks: A hierarchical approach. arXiv **1509.07813**.
