# Supplementary material for: Do-it-yourself networks: a novel method of generating weighted networks
Source: R Soc Open Sci. 2017 Nov 22;4(11):171227. doi: 10.1098/rsos.171227 (PMC5717683; doi:10.1098/rsos.171227)
Supplement: Supplemental Material B. Algorithm source code [file rsos171227supp2.docx]

*Supplemental material B*

*Do-it-yourself (DIY) networks: A novel method of generating weighted networks*

David W. Shanafelt

Kehinde R. Salau

Jacopo A. Baggio

ALGORITHM SOURCE CODE

% Code originally written by Kehinde Salau and Jacopo Baggio

% Updated and commented by David W Shanafelt and Kehinde Salau

% Optimization code to generate adjacency matrices with a given spectral radius, variance of dominant eigenvector, and skewness of dominant eigenvector

% Outputs a corresponding adjacency matrix for use in network generation

%%% Example call - [x,fval,maxeig,EstVar,EstSkew,IfConverge,IfGlobalMin] = NetworkGASkew_weighted(1,20,6,20,0.05,0.25) %%%

%%% Example call - [x,fval,maxeig,EstVar,EstSkew,IfConverge,IfGlobalMin] = NetworkGASkew_weighted(1,20,6,35,0.06,0.6) %%%

% imin - the minimum value of a weighted link

% imax - the maximum value of a weighted link

% n - number of nodes in the network (e.g. 'n' dimensional adjacency matrix)

% lamb - choice value of the spectral radius

% EigVar - choice value for the variance of the eigenvector centrality

% EigSkew - choice value for the skewness of the eigenvector centrality

function [x,fval,maxeig,EstVar,EstSkew,IfConverge,IfGlobalMin] = NetworkGASkew_weighted(imin,imax,n,lamb,EigVar,EigSkew)

%%% Initial conditions (initial adjacency matrix) %%%

x0 = randi(imax,n,n);

x0(1:n+1:n*n) = 0; % makes the matrix zero diagonal

%%% Constraints for the adjacency matrix %%%

% Set up empty matrices for the constraints

% Note that we use the notation of MatLab (see the main text for details)

Aeq = zeros(n^2,n^2); % boundary equality conditions matrix (matrix B in the main text)

Beq = zeros(n^2,1); % equality conditions matrix (solutions to the equations in Aeq) (matrix E in the main text)

lb = zeros(n^2,1); % lower bound for entries in the solution matrix (min values of entries of the adjacency matrix)

ub = zeros(n^2,1); % upper bound for entries in the solution matrix (max values of entries of the adjacency matrix)

%%% Building AEQ (matrix B in the main text) %%%

%%% Step 1 - Identify the entries of AEQ corresponding to the DIAGONAL entries of our adjacency matrix %%%

DiagInd = (1:n+1:n*n); % one-dimensional index of diagonal entries (not limited to a fully connected network)

%%% Step 2 - Identify the entries of AEQ corresponding to the OFF-DIAGONAL entries of our adjacency matrix %%%

OffDiagInd=[];

OffDiagTranInd=[];

for k = 1:n

OffDiagInd = [OffDiagInd DiagInd(k)+1:1:k*n]; % one-dimensional index of the lower, off-diagonal terms

OffDiagTranInd = [OffDiagTranInd DiagInd(k)+n:n:n^2]; % one-dimensional index of the upper, off-diagonal terms

end

OffDiagTotInd = [OffDiagInd OffDiagTranInd]; % all entries of the off-diagonals

%%% Step 3 - Build the 'bounding constraint' matrix (matrix B in the main text) %%%

% The process is highlighted in detail in lines 201-218 in the main text

%%% Step 3a - constrain the diagonals of the adjacency matrix to be zero %%%

for i = 1:n

Aeq(DiagInd(i),DiagInd(i)) = 1; % sets the diagonals that correspond the diagonals of the [n by n matrix]

end

%%% Step 3b - build symmetry into the non-zero, off-diagonal entries of the adjacency matrix %%%

for i = 1:length(OffDiagInd)

Aeq(OffDiagInd(i),OffDiagInd(i)) = 1;

Aeq(OffDiagInd(i),OffDiagTranInd(i)) = -1; % transpose identical and set equal

end

%%% Step 3c - constrain the solutions to lower and upper bounds %%%

for j = 1:length(OffDiagTotInd) % loop through every off-diagonal entry (lower and upper)

lb(OffDiagTotInd(j)) = imin;

ub(OffDiagTotInd(j)) = imax;

end

%% Optimization %%

[maxvec,maxeig] = eigs(x0,1); % calculates (and outputs) the dominant eigvalue and eigenvector for initial adjacency matrix x0

x=x0; % defines adjacency matrix for numerical hill climb method

EigVec = abs(maxvec); % vector associated with the dominant eigenvalue

EstVar = var(EigVec); % variance of EigVec

EstSkew = skewness(EigVec); % skewness of EigVec

tick = 1; % tracker for total number of iterations

TickTol = 100; % total number of iterations allowed before breaking the code

% options for numerical solver

options = optimset('fmincon'); % sets the numerical solver ('interior-point' (default), 'sqp')

options.Algorithm = 'sqp'; % sets the maximizing/minimizing algorithm

% options for error tolerance and number of evaluations of the solver

% options.TolX = 1e-20;

% options.TolCon = 1e-20;

% options.TolFun = 1e-20;

options.MaxFunEvals = n^10 * 10^n; % max number of evaluations of minimizer function

options.MaxIter = n^10 * 10^n; % max number of iterations of the hill-climb method

options = optimset('Display', 'off');

Value = inf; % initial value of minimization function

BadTick = 0; % tracker for iterations w/o convergence to a solution

BadTickTol = 10; % maximum number of 'bad' iterations allowed

GlobalMin = 0; % tracker for testing for a global minimum

GlobalMinTol = 25; % maximum number of iterations allowed before determining a global min

IfConverge = 0; % tracker for if algorithm converged to a solution

IfGlobalMin = 0; % tracker for if algorithm converged to a solution

%%% main iteration %%%

% Numerical hill climb method outlined in lines 219-239 in the main text

% The numerical hill climb method will continue until our solutions are within a particular value or percentage from those desired

% disp('Beginning numerical hill climb method...')

while (abs((maxeig - lamb)/lamb)*100 > 5) || (abs((EstVar - EigVar)/EigVar)*100 > 5) || (abs((EstSkew - EigSkew)/EigSkew)*100 > 5);

% while (abs((maxeig - lamb)/lamb) > 1e-5) || (abs((EstVar - EigVar)/EigVar) > 1e-5) || (abs((EstSkew - EigSkew)/EigSkew) > 1e-5);

if(tick >= TickTol) % if exceed max # iterations, break

clc

disp('We''ve died of dysentery')

break

elseif (BadTick >= BadTickTol)

x0 = randi(imax,n,n); % reset initial matrix

x0(1:n+1:n*n) = 0; % makes the matrix zero diagonal

clc

disp('new initial conditions!')

BadTick = 0;

end

x0 = x; % defines the 'old' adjacency matrix as the current initial conditions

% defines 'old' adjacency matrix and variables

oldx = x; % really define 'old' x

oldL = maxeig;

oldV = EstVar;

oldS = EstSkew;

% redefine 'new' adjacency matrix (perturbing an (or set of) off-diagonal entry(ies) of x0)

% x0(OffDiagTotInd(randi(length(OffDiagTotInd)))) = randi(imax,1); % replaces one off-diagonal entry

x0(OffDiagTotInd( randi(length(OffDiagTotInd),ceil(n/2),1) )) = randi(imax,1); % replaces no more than ~1/4 of off-diagonal entries

% optimizer - outputting x values and the (minimum) value of the objective function

[x,fval] = fmincon(@(x)objecfunSkew_weighted(n,x,lamb,EigVar,EigSkew),x0,[],[],Aeq,Beq,lb,ub,[],options);

if(fval > Value) % checks to make sure our minimizer keeps minimizing (fval decreases with the iterations)

% if (abs(fval - Value)/Value >= 0.01)

% if (fval - Value >= 1e-5)

disp('Bad perturbation')

BadTick = BadTick+1;

x = oldx; % define the perturbed adjacency matrix as new initial conditions

tick = tick + 1; % adds one to number of iterations

continue % moves forward in the hill climb method

else

disp('Good perturbation')

[maxvec,maxeig] = eigs(x,1); % eigenvalues of the new matrix

% defines 'current' properties of adjacency matrix

% EigVec = abs(maxvec);

EigVec = maxvec;

EstVar = var(EigVec);

EstSkew = skewness(EigVec);

% determines if the numerical algorithm gets 'stuck' in the optimization process

if (abs(oldL - maxeig) <= 1e-10) && (abs(oldV - EstVar) <= 1e-10) && (abs(oldS - EstSkew) <= 1e-10)

if GlobalMin > GlobalMinTol

break

end

x0 = randi(imax,n,n); % reset initial matrix

x0(1:n+1:n*n) = 0; % makes the matrix zero diagonal

clc

disp('Gaaaaah!!! New initial conditions!')

GlobalMin = GlobalMin + 1 % adds one to tracker for global min

end

% redefine value of objective function

Value = fval;

BadTick = 0;

% display iteration, value of minimization function, and eigenmetrics

disp(['iterations = ' num2str(tick)])

disp([num2str(fval) ' (fval) ' num2str(maxeig) ' (lambda) ' num2str(EstVar) ' (variance) ' num2str(EstSkew) '(skewness) '])

end

tick = tick + 1; % adds one to number of iterations

end

% indicator variable for if we converged to a solution

if (abs((maxeig - lamb)/lamb)*100 < 5) && (abs((EstVar - EigVar)/EigVar)*100 < 5) && (abs((EstSkew - EigSkew)/EigSkew)*100 < 5)

IfConverge = 1;

end

% indicator for if the numerical algorith got 'stuck'

if GlobalMin > GlobalMinTol

IfGlobalMin = 1;

end

function f = objecfunSkew_weighted(n,x,lamb,EigVar,EigSkew)

% General algorithm to read in/convert the initial x from a vector to a matrix

% We need to make sure it is converted to a matrix before we can calculate its eigenmetrics

% x = vec2mat(x,n); % converts to matrix

[V,D] = eigs(x,1); % pulls dominant eigenvalue (D) and eigenvector (D) from matrix x

% EigVec = abs(V); % absolute value of the eigenvector associated with the dominant eigenvalue

EigVec = V; % absolute value of the eigenvector associated with the dominant eigenvalue

EstVar = var(EigVec); % variance

EstSkew = skewness(EigVec); % skewness

w1 = 10^4; % weight associated with spectral radius (10^4)

w2 = 10^7; % weight associated with variance of eigenvector (10^7)

w3 = 10^6; % weight associated with skewness of eigenvector (10^6)

% function that we are minimizing: (sum of squared differences between the desired and estimated eigenmetrics)

f = w1*(D - lamb)^2 + w2*(EstVar - EigVar)^2 + w3*(EstSkew - EigSkew)^2;

% Note that variance and skewness are relatively small numbers compared to the spectral radius (dominant eigenvalue). We need to weight them so

% that the algorithm minimizes those distances.
